# Supplementary material for: P-TRAP: a Panicle Trait Phenotyping tool
Source: BMC Plant Biol. 2013 Aug 29;13:122. doi: 10.1186/1471-2229-13-122 (PMC3848748; doi:10.1186/1471-2229-13-122)
Supplement: Additional file 7 — P-TRAP source code. The Netbeans project that contains the source code of the application. [file 1471-2229-13-122-S7.zip › javadoc.zip!/javadoc/org-bioalg-skeletonprocessor/org/bioalg/skeletonprocessor/SkeletonExteractor.html]

SkeletonExteractor (SkeletonProcessor)


JavaScript is disabled on your browser.


- Package
- Class
- Tree
- Deprecated
- Index
- Help

*org.bioalg.skeletonprocessor 1.0*

- Prev Class
- Next Class

- Frames
- No Frames

- All Classes

- Summary:
- Nested |
- Field |
- Constr |
- Method

- Detail:
- Field |
- Constr |
- Method


org.bioalg.skeletonprocessor

## Class SkeletonExteractor

- java.lang.Object
- - org.bioalg.skeletonprocessor.SkeletonExteractor

- ---

    

  ```
  public class SkeletonExteractor
  extends java.lang.Object
  ```

  Zhang-Suen algorithm in two different implementation.
  Zhang, T. Y., and Ching Y. Suen.
  "A fast parallel algorithm for thinning digital patterns." Communications of the ACM 27.3 (1984): 236-239.

  Author:
  :   faroq

- - ### Field Summary

    Fields

    | Modifier and Type | Field and Description |
    | --- | --- |
    | `static int` | `NORTH` |
    | `static int` | `SOUTH` |
  - ### Constructor Summary

    Constructors

    Constructor and Description

    `SkeletonExteractor(org.bioalg.imageprocessor.AImage image)`
  - ### Method Summary

    Methods

    | Modifier and Type | Method and Description |
    | --- | --- |
    | `int` | `calc01occurance(int[][] mat)` |
    | `int` | `calcNonzero(int[] vector)` calculate the number of nonzero pixels. |
    | `int` | `calcNonzero(int[][] mat)` |
    | `int` | `calcNonzero(int[][] mat, int ksize)` |
    | `void` | `clean(int n)` |
    | `void` | `cleanimage(int ksize, int minimunpixels)` |
    | `void` | `deletepixels()` |
    | `int` | `firstsubiteration(int n)` |
    | `org.bioalg.imageprocessor.AImage` | `getImage()` |
    | `int[][]` | `getNbyNsubmatrix(int i, int j, org.bioalg.imageprocessor.AImage im, int n)` return nXn matrix of the image |
    | `int[]` | `getNvector(int i, int j, org.bioalg.imageprocessor.AImage im, int n)` |
    | `void` | `hotstair(int dir)` |
    | `void` | `removesubmatrix(int n, int i, int j)` |
    | `void` | `setImage(org.bioalg.imageprocessor.AImage image)` |
    | `void` | `skeletonizeEXSZ()` Based on table implementation found here: http://rsbweb.nih.gov/ij/developer/source/ij/process/BinaryProcessor.java.html. |
    | `void` | `skeletonizeSZ()` The implementation of the SZ method based on the article specification. |
    | `int` | `tableBasedSZThining(int pass)` Based on table implementation found here: http://rsbweb.nih.gov/ij/developer/source/ij/process/BinaryProcessor.java.html. |

    - ### Methods inherited from class java.lang.Object

      `clone, equals, finalize, getClass, hashCode, notify, notifyAll, toString, wait, wait, wait`

- - ### Field Detail


    - #### NORTH

      ```
      public static int NORTH
      ```


    - #### SOUTH

      ```
      public static int SOUTH
      ```
  - ### Constructor Detail


    - #### SkeletonExteractor

      ```
      public SkeletonExteractor(org.bioalg.imageprocessor.AImage image)
      ```
  - ### Method Detail


    - #### getImage

      ```
      public org.bioalg.imageprocessor.AImage getImage()
      ```


    - #### setImage

      ```
      public void setImage(org.bioalg.imageprocessor.AImage image)
      ```


    - #### skeletonizeEXSZ

      ```
      public void skeletonizeEXSZ()
      ```

      Based on table implementation found here: http://rsbweb.nih.gov/ij/developer/source/ij/process/BinaryProcessor.java.html.
      The code here is ported to support our classes, we would like here to note that as there is not paper to cite for this table-based method
      Please cite the imagej paper (not the P-TRAP article), in case you want to use only this function or any calls inside it.


    - #### skeletonizeSZ

      ```
      public void skeletonizeSZ()
      ```

      The implementation of the SZ method based on the article specification.

      See Also:
      :   `skeletonizeEXSZ()`


    - #### clean

      ```
      public void clean(int n)
      ```


    - #### deletepixels

      ```
      public void deletepixels()
      ```


    - #### firstsubiteration

      ```
      public int firstsubiteration(int n)
      ```


    - #### removesubmatrix

      ```
      public void removesubmatrix(int n,
                         int i,
                         int j)
      ```


    - #### getNbyNsubmatrix

      ```
      public int[][] getNbyNsubmatrix(int i,
                             int j,
                             org.bioalg.imageprocessor.AImage im,
                             int n)
      ```

      return nXn matrix of the image

      Parameters:
      :   `i` -
      :   `j` -
      :   `im` -
      :   `n` -

      Returns:
      :   nXn submatrix


    - #### getNvector

      ```
      public int[] getNvector(int i,
                     int j,
                     org.bioalg.imageprocessor.AImage im,
                     int n)
      ```

      Parameters:
      :   `i` -
      :   `j` -
      :   `im` -
      :   `n` -

      Returns:
      :   an n\*n vector of an image given a point (i,j), the vector is 1D
          of nbyn matrix


    - #### calcNonzero

      ```
      public int calcNonzero(int[] vector)
      ```

      calculate the number of nonzero pixels.

      Parameters:
      :   `vector` -

      Returns:


    - #### calcNonzero

      ```
      public int calcNonzero(int[][] mat)
      ```


    - #### calcNonzero

      ```
      public int calcNonzero(int[][] mat,
                    int ksize)
      ```


    - #### calc01occurance

      ```
      public int calc01occurance(int[][] mat)
      ```


    - #### hotstair

      ```
      public void hotstair(int dir)
      ```


    - #### cleanimage

      ```
      public void cleanimage(int ksize,
                    int minimunpixels)
      ```


    - #### tableBasedSZThining

      ```
      public int tableBasedSZThining(int pass)
      ```

      Based on table implementation found here: http://rsbweb.nih.gov/ij/developer/source/ij/process/BinaryProcessor.java.html.
      The code here is ported to support our classes, we would like here to note that as there is not paper to cite for this table-based method
      please cite the imagej paper (not the P-TRAP article), in case you want to use only this function or any calls inside it. We claim no ownership of this part of the code except the porting efforts only.

      Parameters:
      :   `pass` - is the number of the current pass


- Package
- Class
- Tree
- Deprecated
- Index
- Help

*org.bioalg.skeletonprocessor 1.0*

- Prev Class
- Next Class

- Frames
- No Frames

- All Classes

- Summary:
- Nested |
- Field |
- Constr |
- Method

- Detail:
- Field |
- Constr |
- Method
